# Functional network organization is locally atypical in children, adolescents, and young adults with congenital heart disease

**DOI:** 10.1016/j.nicl.2026.103965

**Published:** 2026-02-13

**Authors:** Joy Roy, William Reynolds, Julia Wallace, Daryaneh Badaly, Rafael Ceschin

**Affiliations:** a Department of Biomedical Informatics, University of Pittsburgh, School of Medicine, United States; b Department of Radiology, UPMC Children’s Hospital of Pittsburgh, United States; c Learning and Development Center, Child Mind Institute, New York, NY, United States

**Keywords:** Functional connectivity, Network science, Congenital heart disease

## Abstract

Children, adolescents, and young adults with congenital heart disease (CHD) frequently experience disruptions in neurodevelopment affecting their executive functioning and other cognitive abilities, which in turn can impact academic performance, psychosocial adjustment, and overall quality of life. This exploratory study aims to investigate the impact of CHD on functional brain network connectivity and cognitive function, with a particular focus on executive functioning. Rather than relying on a single network construction method or arbitrary thresholds, our study methodically employed both weighted networks and binarized networks generated using absolute and proportional thresholding. This cross-method approach enables us to identify functional connectivity features that persist across heuristically and arbitrarily defined parameters, and to evaluate their association with neurocognition. Using resting-state fMRI data, we examined several network metrics across brain regions using three network construction types: weighted networks, absolute-threshold binarized networks, and proportional-threshold binarized networks. Regression models were then fit to neuropsychological test scores using metrics obtained from each network construction approach. Our results identified differences in network connectivity with a predilection for temporal, occipital, and subcortical regions, across both weighted and binarized networks. Furthermore, we identified distinct correlations between network metrics and cognitive performance, suggesting potential compensatory mechanisms within specific brain regions. These results provide an initial, methodologically transparent characterization of altered network organization in CHD and offer directions for future hypothesis-driven investigations.

## Highlights

• CHD youth show altered brain networks linked to executive function deficits.
• CHD shows temporal, occipital, and subcortical network changes tied to cognition.
• Local network differences were more prominent than global differences.
• This study used a cross-method network analysis to avoid reliance on arbitrary thresholds.

## 1 Introduction

Congenital heart disease (CHD) is a broad term for congenital anomalies affecting the heart's structure and function, evident from birth (Gonzalez et al., 2021, Sun et al., 2015). It stands as one of the most prevalent congenital defects, impacting nearly 1 in 100 infants annually in the United States (Sun et al., 2015). Studies have consistently shown that individuals with CHD are more likely to struggle with school performance and psychosocial adjustment early in life, and may encounter obstacles in their careers later on, such as job-related mobility difficulties (Kamphuis et al., 2002, Schaefer et al., 2016, Warnes et al., 2008). Individuals born with CHD face an increased risk of developing cognitive impairments compared to term-born, healthy peers, with differences seen across a number of domains including language skills, visuospatial skills, attention and executive functioning, and learning and memory (Nattel et al., 2017, Russell et al., 2018). In turn, cognitive impairments are thought to underlie the academic, adaptive, and adjustment vulnerabilities that those with CHD face. Given the disruptions in cognitive functioning seen in CHD and their importance for adjustment throughout an individual’s life, identifying cohort-specific neurostructural and neurofunctional correlates of abnormal development is imperative for mitigating negative downstream outcomes affecting overall quality of life by facilitating better prognosis and treatment planning.

There is a strong body of evidence showing disrupted brain connectivity as a central mechanism linking cardiac pathophysiology to neurocognitive outcomes. Our lab has previously demonstrated that aberrant functional connectivity is present in newborns with CHD before cardiac surgery (Votava-Smith et al., 2022) indicating that altered cerebral hemodynamics during fetal development already impact brain organization. This is similarly shown by De Asis-Cruz et al (De Asis-Cruz et al., 2018). Ni Bhroin et al. (2020) identified reduced structural connectivity in the cortico-striatal-thalamic network, which is critical for motor control, executive function, and emotional regulation (Ni Bhroin et al., 2020). This suggests that subcortical-cortical circuits are particularly vulnerable to altered oxygen delivery and hemodynamic instability. In early development, Bonthrone et al. (2022) found that neonatal frontal-limbic connectivity patterns predict externalizing behaviors (aggression, impulsivity) in toddlerhood, establishing early brain-behavior relationships (Bonthrone et al., 2022). By preschool, white matter network topology has been shown to be disrupted in children with tetralogy of Fallot, indicating that abnormal vascular perfusion continues to affect brain development during critical periods of myelination and synaptic pruning (Liu et al., 2024). Taken together, we observe a pattern of cumulative clinical risk throughout development (Ehrler et al., 2024).

In this work, we focused our models on executive functioning skills. Executive functions are a set of cognitive skills needed for goal directed action that include, for example, working memory (or the ability the mentally manipulate information in mind), inhibitory control (or the ability to stop prepotent draws for attention, thoughts, and behaviors), and mental flexibility (or the ability to shift between different streams of information, task demands, and responses) (Diamond, 2013). Executive functioning skills show a prolonged period of maturation with notable developments seen through the school-age period and into young adulthood. Deficits in executive functioning have been linked to poorer adjustment in adolescence and young adulthood, both across the population and specifically for those with CHD (Diamond, 2013, Gerstle et al., 2016, Sanz et al., 2018). Given their role in regulating other cognitive skills, their developmental trajectory, and their importance throughout life, executive functions offer an interesting set of skills to explore functional brain networks.

Functional connectivity (FC) aims to identify linear temporal correlations between blood oxygen level dependent (BOLD) signals between pair-wise spatial regions of the brain (Filippi et al., 2019, Mohanty et al., 2020, Smitha et al., 2017). FC analyses yield networks, with nodes representing brain regions and edges representing functional correlations between regions. Applying graph analysis metrics to these networks provides insights into underlying models of connectivity (Filippi et al., 2019, Mohanty et al., 2020, Smitha et al., 2017). While these metrics are generally subject matter agnostic, they contribute to a deeper understanding of brain-specific connectivity patterns. Numerous studies have explored FC in relation to neurodevelopmental, neurological, neurodegenerative, and psychiatric diseases, identifying connectivity differences to typically-developing and normative samples (Filippi et al., 2019, Ingalhalikar et al., 2021, Mohanty et al., 2020, Smitha et al., 2017).

When constructing network matrices, the choice between weighted and binary networks has downstream effects on network metric values and interpretations. Weighted networks are less commonly used, but more directly constructed; they assign the raw correlation value between nodes to the edges, leading to maximally large, more complex networks. Binarization, while simplifying network architecture, inevitably sacrifices information by converting continuous values into an on/off representation (Korhonen et al., 2021). Binary networks, nevertheless, offer distinct advantages in pruning edges and simplifying complex connectivity patterns by clearing potentially weak or indirect second-order correlations. Absolute thresholding, a method involving the setting of a fixed-valued correlation strength, preserves edges above this threshold while discarding others. In contrast, proportional thresholding retains a fixed percentage of the strongest edges, ensuring consistent density across subject matrices. Each approach presents trade-offs; weighted thresholding considers the impact of weak edges on network topology, known to influence network topology and dynamics (Korhonen et al., 2021, Santarnecchi et al., 2014). While weighted networks overcome the need for arbitrary thresholds, they are more susceptible to signal-to-noise ratio (SNR) fluctuations. Absolute thresholding ensures a minimum connection strength among all patients but may lead to varying edge numbers between individuals and groups, potentially causing erroneous distinctions between groups (Korhonen et al., 2021). Proportional thresholding addresses this issue by controlling for density or network cost, though it does not account for connection strength (Achard and Bullmore, 2007, van den Heuvel et al., 2017). Finally, there is no consensus in the literature indicating optimal threshold selection, and ranges or heuristically selected thresholds are often selected (Garrison et al., 2015).

In this study, we investigated FC in young persons with CHD using graph analysis metrics. We examined the relationships between FC and executive function, with a focus on three core skills (i.e., working memory, inhibitory control, and mental flexibility). Our goal was to explore differences in brain connectivity between CHD patients and a healthy sample as well as the associated links with executive functioning, in order to better understand functional reorganization associated with CHD and its impact. Recognizing the challenges in network and threshold selection, our study deliberately employed both weighted and binarized networks, focusing on identifying robust FC signals that persist across heuristically and arbitrarily defined parameters, and investigated their comparative results.

## 2 Methods

### 2.1 Participants

**Fig. 1:**
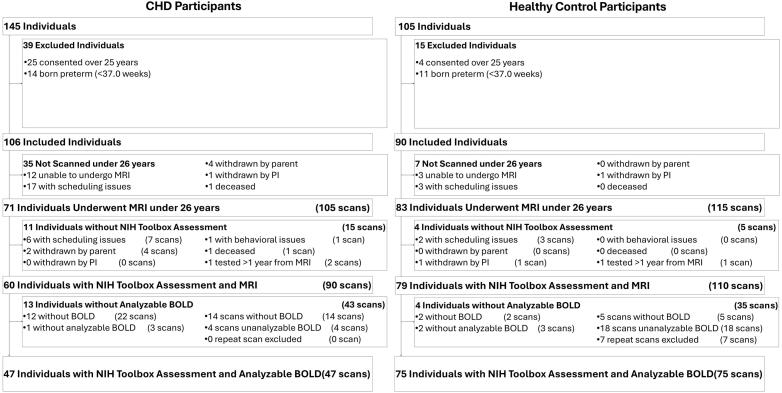
Patient Inclusion and Exclusion Flowchart: The initial dataset comprised 250 patients. Patients were excluded based on the following criteria: age over 25 years, preterm birth, absence of an NIH Toolbox assessment, or lack of analyzable BOLD data. After applying these criteria, the final cohort included 47 individuals with congenital heart disease (CHD) and 75 control patients, totaling 122 participants.

Participants were prospectively recruited from a single institution. Our exclusion criteria included comorbid genetic disorders, contraindications for magnetic resonance imaging (MRI) (e.g., a pacemaker), non-English speakers (who could not complete our neuropsychological battery), and age under 6 years or over 25. For healthy controls, study exclusion criteria also included preterm birth and neurological abnormalities (e.g., brain malformations, history of stroke, hydrocephalus). Patients with CHD included a heterogenous mix of cardiac lesions, including hypoplastic left heart syndrome (HLHS), aortic arch abnormalities, d-transposition of the great aorta (d-TGA), and other malformations requiring surgical correction in the first year of life. 145 patients with CHD and 105 healthy controls were initially screened. 71 patients with CHD and 83 controls successfully underwent MRI. For this analysis, we excluded individuals with less than 4.5 min of usable single-sequence BOLD scan time. All patient scans were within a year of neuropsychological testing. If a patient had multiple scans, due to clinical follow-up or repeat acquisitions, we used the scan closest to the date of neuropsychological testing. Of the remaining sample, 47 patients with CHD and 75 controls completed neuropsychological testing and had analyzable BOLD scans. Fig. 1 shows the inclusion and exclusion flowchart and the resulting number of participants retained from each criterion. Patients were recruited with Institutional Review Board (IRB) approval and oversight (University of Pittsburgh Institutional Review Board STUDY20060128 Multimodal Connectome Study, approval 23 July 2020, and STUDY1904003 Ciliary Dysfunction, Brain Dysplasia, and Neurodevelopmental Outcome in Congenital, approval 6 February 2023). Written informed consent was obtained from all participants or their parents, as relevant, and assent was collected from all minor individuals in the study. We have published previous analyses of this cohort (Lee et al., 2023, Sahel et al., 2023, Schmithorst et al., 2022a, Schmithorst et al., 2022b, Wallace et al., 2023).

### 2.2 Preprocessing

**Fig. 2:**
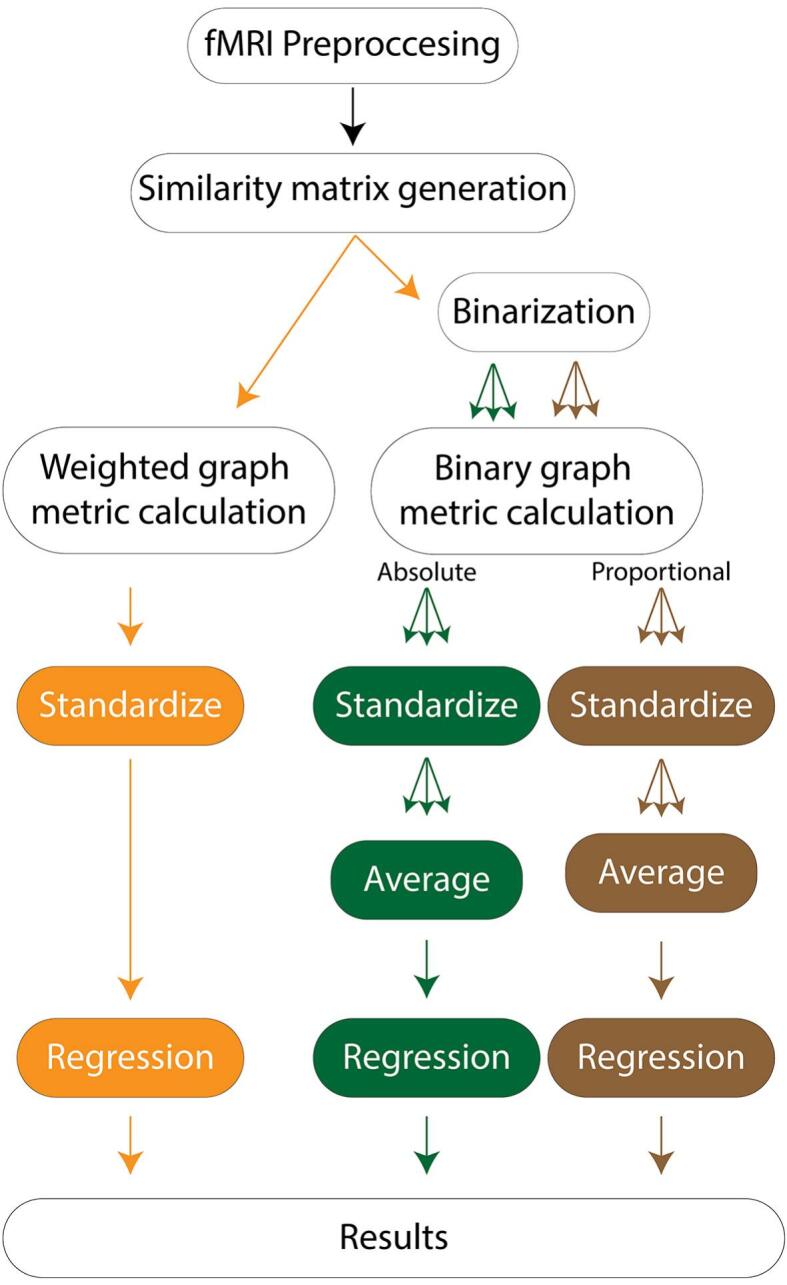
Study summary workflow. Study summary workflow. First, each patient’s fMRI scan underwent preprocessing and scrubbing in accordance with the guidelines outlined in Power et al. (2014). Next, similarity matrices were constructed using the absolute value of the Pearson correlation between regions defined by the Automated Anatomical Labeling (AAL) template. These matrices were then used for both weighted analyses and for binarization in binary analyses, applying absolute and proportional thresholds iteratively across a range of values. After thresholding, graph metrics were computed for each metric and each brain region and standardized across participants. All graph-metric computations, standardization, averaging, and regression analyses were performed on a within-region basis. Finally, for the primary analyses, threshold-specific values were averaged within each region and metric to produce a single summary value per participant brain region, which was then used as input to regression models predicting neurocognitive outcomes.

Fig. 2 shows our pre- and post-processing workflow. Scans underwent preprocessing through a customized pipeline developed using the FMRIB Software Library (FSL) and Python 3.6 (Jenkinson et al., 2012). This approach follows previously published BOLD motion correction and quality-control guidelines (Power et al., 2014). The preprocessing included motion correction, skull stripping, and normalization. Additionally, images underwent a 6 mm Full Width at Half Maximum (FWHM) spatial smoothing filter and temporal bandpass filtering (0.009 Hz < f < 0.08 Hz). The subsequent steps included regression of motion correction realignment parameters and their first derivatives. Frames with framewise displacement (FD) > 0.5 mm and derivative of the temporal variance (DVARS) > 5 were censored and scrubbed before similarity matrix calculation, ensuring a minimum of 4.5 min of usable data for all patients. The MNI152 template was registered into patient space using linear followed by nonlinear transformations, and segmentation utilized a modified version of the Automated Anatomical Labeling atlas (AALv3). To address reliability concerns in registration in regions with limited number of voxels, thalamic subdivisions were amalgamated into whole thalamic regions, maintaining laterality distinctions. Additionally, regions with fewer than 20 voxels were excluded from further analysis (i.e., left and right locus coeruleus, left and right ventral tegmental area, and dorsal and medial raphe nucleus).

### 2.3 Network construction

Following preprocessing, the average intensity value for each brain region was computed per timepoint. Pearson correlation coefficients were then calculated between each pair of brain regions over time for each patient, resulting in a patient-specific N x N matrix. Each row and column in this matrix represented a brain region, and each cell held the degree of similarity between the regional temporal BOLD signals. To preserve both strong positive and negative correlations, all matrix values were converted to their absolute values. Subsequently, this square matrix was utilized to construct the adjacency matrix for each patient's brain network. Our analysis incorporated both a weighted approach and a binary approach, with both absolute and proportional thresholding. The raw matrix served as input for weighted network computations.

To address the arbitrariness of threshold choice in binarization, we adopted an iterative approach, exploring thresholds from 0 to 1 with a step size of 0.05. Constraints were then applied to threshold ranges for both absolute and proportional binarizations to enforce biologically meaningful results. Absolute thresholding had a lower bound threshold of 0.10 to mitigate spurious connections and an upper bound threshold of 0.65 to limit the number of components in the network to where the mean and median number of components in the network were less than 2. Proportional thresholding ranged from 0.25, to limit the number of components, to 0.65, where all networks became fully connected (Supplemental Table 1).

### 2.4 Network measure analyses

We used Brain Connectivity Toolbox for Python’s (BCTpy) for graph analysis. BCTpy is specifically designed for examining network properties in structural and functional connectivity (Rubinov & Sporns, 2010). In this study, we employed BCTpy 0.5.2 to compute graph metrics encompassing both weighted and binarized calculations. Nodal efficiency and small world sigma coefficients were not available in BCTpy, and were manually implemented with following equations (1) and (2), respectively. Across patient networks, a comprehensive set of 13 network measures was computed. Global metrics included global efficiency, assortativity, density, modularity, transitivity, and small-world sigma. Regional metrics were local efficiency, nodal efficiency, clustering coefficient, node betweenness, degree, eigenvector centrality, and participation coefficient. All metrics were calculated per patient network across all thresholds.

Nodal Efficiency: where N is the total number of nodes, and i and j represent individual nodes.

(1) $E_{\mathrm{nodal}}\left(i\right)=\frac{1}{N-1}\sum_{j\in G}\frac{1}{d_{\mathrm{ij}}}$

Small World Sigma: where C is the clustering coefficient of the observed network, L is the average path length of the observed network, and Cr and Lr are the clustering coefficient and average path length in comparable random networks, respectively.

(2) $\sigma =\frac{C/C_{r}}{L/L_{r}}$

#### 2.4.1 Global network metrics

Global Efficiency quantifies how efficiently information is exchanged across the entire network. Assortativity indicates the tendency of nodes to connect with others of similar degree. Density reflects the proportion of realized connections to potential connections in the network. Modularity identifies densely connected groups of nodes, revealing community structures. Transitivity measures the degree to which nodes tend to form clusters or triangles. Small World Sigma assesses the balance between segregation and integration in a network, indicating how well it maintains overall connectivity while supporting localized clustering.

#### 2.4.2 Regional network metrics

Regional metrics include Local Efficiency, gauging how efficiently information is exchanged within the immediate neighborhood of a node, and Nodal Efficiency, identifying nodes crucial for the network's overall efficiency. Clustering Coefficient is related to how connected the network is around a particular node. Node Betweenness quantifies the importance of a node in connecting different parts of the network. Degree quantifies the number of connections each node has. Eigenvector Centrality identifies nodes that connect to other well-connected nodes. Participation Coefficient assesses the diversity of a node's connections across different network modules.

### 2.5 Executive function and network connectivity

We applied a regression model to predict each neuropsychological test score, including the following covariates: age, gender, presence of CHD, regional network metric, and the interaction between CHD and regional network metric. As previously discussed in prior analyses of this cohort (Lee et al., 2023, Wallace et al., 2023), participants completed batteries of computerized and face-to-face measures of cognition. For the current analyses, we focused on measures of three core executive functions, with tasks from the National Institute of Health Toolbox (NIHTB) and the Wechsler Intelligence Scale for Children, Fourth Edition/Fifth Edition (WISC-IV/V) (only for those ages 6 to 16) as well as parent-completed ratings from the Behavior Rating Inventory of Executive Function, Second Edition (BRIEF-2) (only for those ages 6 to 18) (Table 2). All neuropsychological outcomes were analyzed as age-corrected standard scores, as provided by the respective test publishers.

For working memory, data was collected using the NIHTB List Sorting Working Memory (LSWM), WISC-IV/V Digit Span, and the BRIEF-2 Working Memory; for inhibitory control, data were collected using NIHTB Flanker Inhibitory Control and Attention (FICA) and the BRIEF-2 Inhibit; for mental flexibility, data were collected using the NIHTB Dimensional Change Card Sort (DCCS) and BRIEF-2 Shift. Although the dataset included additional tests of executive functioning, we selected the measures with the highest rates of complete data. Across cognitive measures, higher numeric scores indicate better performance, with the exception of the BRIEF-2 scales, in which higher scores reflect poorer functioning. Additionally, two composite scores from the NIH Toolbox were included in our analyses: the NIHTB Fluid Composite and NIHTB Crystallized Composite. The former is conceptualized as a measure of fluid cognition and largely includes measures requiring executive functioning, while the latter is an index of crystallized cognition and can serve as a baseline measure independent of executive functioning. While we focused our analyses on executive functions, we included the global metrics of cognition to provide a more holistic understanding of cognitive abilities and complement our focal emphasis.

Each test and regional network metric were analyzed separately. Recognizing the diverse range of values for each network metric (e.g., node betweenness ranges from 0 to 1, while node degree spans from 0 to the total number of nodes in the graph), we opted to standardize each of the metrics across patients before inputting them into the regression model. This standardization process was repeated for both weighted network metrics and for every absolute and proportional thresholded network metric. We then averaged the standardized metric values per region across all thresholds before inputting them into the regression model, as done by Ehrler et al. (Ehrler et al., 2024). Statistical inference for the interaction effect was based on a fixed significance threshold of p < 0.001. In summary, we calculated regression models from three different types of inputs: 1) metrics derived from weighted networks, 2) metrics derived and averaged from a fixed range of absolute thresholds, and 3) metrics derived and averaged from a fixed range of proportional thresholds. All analyses used the following regression model:

NeuropsychologicalScore∼Age+Sex+CHD+GraphMeasure+CHD×GraphMeasure

### 2.6 Ad-Hoc sub-analysis for age groups

FMRI functional connectivity (FC) has been shown to predict biological age of children and young adults 8–22 (Dosenbach et al., 2010, Gu et al., 2015). To investigate the effects of the large age range within our cohorts, we ran an ad-hoc analysis using two age-based stratifications: 8–11 years and 12 + years. These cut points were selected to align with how the NIH Toolbox is normed and stratified. We restricted these supplemental analyses to the NIH Flanker Inhibitory Control and Attention Test, NIH DCCS, and NIH Fluid Composite, as these were the domains with sufficient sample sizes to support meaningful stratification. The number of participants in each stratification is shown in Supplemental Table 2.

### 2.7 Ad-Hoc sub-analysis for system segregation

Because alterations in CHD may disproportionately affect regions with high metabolic demand and strong hub-like properties, we conducted an exploratory sub-analysis examining system segregation within the Default Mode Network (DMN). We computed the mean functional connectivity within the DMN subnetwork (Z_w_) and the mean functional connectivity between the DMN and all external regions (Z_b_), then used these values to calculate the system segregation index.

$$\mathrm{SystemSegregation}=\frac{\overset{¯}{Z_{w}}-\overset{¯}{Z_{b}}}{\overset{¯}{Z_{w}}}$$

We then modeled the effect of CHD on this segregation measure:

$$SystemSegregation\left(DMN\right)\;∼\;\;Age+Sex+CHD$$

System Segregation was defined following Chan et al. (2014), where the metric reflects the difference between mean within-system and mean between-system correlations, expressed as a proportion of the mean within-system correlation (Chan, Park, Savalia, Petersen, & Wig, 2014).

This study uses the AALv3 atlas, and the following labels were selected to define the Default Mode Network (DMN): 'Frontal_Sup_Medial_L', 'Frontal_Sup_Medial_R', 'Cingulate_Post_L', 'Cingulate_Post_R', 'Angular_L', 'Angular_R', 'Precuneus_L', 'Precuneus_R', 'ACC_sub_L', 'ACC_sub_R', 'ACC_pre_L', 'ACC_pre_R', 'ACC_sup_L', 'ACC_sup_R'. These regions were informed by Oliver et al. (2019), who defined the DMN using AALv2 labels (Oliver, Hlinka, Kopal, & Davidsen, 2019). In their parcellation, the region labeled “Cingulum Anterior” appears as a single parcel; however, in AALv3 this region is subdivided into three distinct parcels (ACC_sub, ACC_pre, and ACC_sup). Accordingly, we used the AALv3 subdivisions in place of the single AALv2 label to maintain anatomical accuracy.

## 3 Results

### 3.1 Participant characteristics and cognitive performance

No statistically significant differences in age, race, ethnicity, and Child Opportunity Index (COI; a measure of socioeconomics) were observed between CHD and control participants. Compared to controls, CHD patients included a greater proportion of males and had lower levels of maternal education. Patient demographics by group are summarized in Table 1.

**Table 1:** Patient Demographics.

Characteristics	CHD (n = 47)	Control (n = 75)	Chi-Square / T-Test p
Males, No. (%)	29 (61.70%)	35 (46.67%)	0.106
Race			0.060
White, No. (%)	40 (85.11%)	48 (64.00%)	
Black or African American, No. (%)	2 (4.26%)	12 (16.00%)	
Asian, No. (%)	0 (0.00%)	2 (2.67%)	
Biracial, No. (%)	5 (10.64%)	13 (17.33%)	

Ethnicity			0.071
Hispanic/Latino	0 (0.00%)	5 (6.67%)	
Non-Hispanic/Latino	47 (100%)	70 (93.33%)	

Age at MRI Scan(s)			0.419
No. (%)	47 (100%)	75 (100%)	
mean (SD)	14.59 (4.39)	13.95 (4.12)	
range	6.59–––25.43	6.11–––21.80	
median (Q1, Q3)	14.40 (11.32, 16.94)	13.85 (11.11, 16.14)	

Highest Maternal Education Provided			0.025
No. (%)	42 (89.36%)	67 (89.33%)	
mean (SD)	1.71 (0.46)	1.81 (0.50)	
range	1.00–––2.00	0.00–––2.00	
median (Q1, Q3)	2.00 (1.00, 2.00)	2.00 (2.00, 2.00)	
Education Not Provided, No. (%)	5 (10.64%)	8 (10.67%)	
No HS Degree, No. (%)	0 (0.00%)	3 (4.00%)	
HS Degree/Some College, No. (%)	12 (25.53%)	7 (9.33%)	
College Degree, No. (%)	30 (63.83%)	57 (76.00%)	
Other College Degree, No. (%)	4 (8.51%)	6 (8.00%)	
Trade/Technical/Vocational Training, No. (%)	2 (4.26%)	2 (2.67%)	
Associates Degree, No. (%)	7 (14.89%)	8 (10.67%)	
Bachelor's Degree, No. (%)	7 (14.89%)	23 (30.67%)	
Master's Degree, No. (%)	6 (12.77%)	13 (17.33%)	
Professional Degree, No. (%)	3 (6.38%)	2 (2.67%)	
Doctorate Degree, No. (%)	1 (2.13%)	3 (4.00%)	

COI Nationally-Normed Overall Score			0.951
No. (%)	47 (100%)	75 (100%)	
mean (SD)	63.26 (26.18)	62.93 (28.93)	
range	3.00–––100.00	10.00–––99.00	
median (Q1, Q3)	64.00 (45.00, 89.50)	65.00 (40.00, 95.50)	
Very Low	2 (4.26%)	10 (13.33%)	
Low	6 (12.77%)	10 (13.33%)	
Moderate	12 (25.53%)	13 (17.33%)	
High	14 (29.79%)	18 (24.00%)	
Very High	13 (27.66%)	24 (32.00%)	

Notes. Maternal Education: 0 = No HS Diploma, 1 = HS Diploma/Some College, 2 = College Degree. COI = Childhood Opportunity Index.

There were group differences for measure of working memory but not other areas of executive functioning. Cognitive test scores for both cohorts are listed in Table 2. Note larger numeric scores in the cognitive measures indicate better performance, with the exception of larger numeric scores on the BRIEF-2 scores indicating poorer ratings of functioning. BRIEF-2 scores showed bias towards high- and normative-performing participants (Supplemental Fig. 3), indicating skewness and a deviation from normality.

**Table 2:** Differences in Neuropsychological Scores between CHD and Control. *Note: Higher scores indicate better performance on all measures except the BRIEF-2, where higher scores correspond to poorer functioning.*

Neurodevelopmental Assessment Outcomes	CHD (n = 47)	Control (n = 75)	T-Test p
**NIH Toolbox Cognitive Battery** Age at Completion, y			0.413
No. (%)	47 (100%)	75 (100%)	
mean (SD)	14.58 (4.39)	13.94 (4.10)	
range	6.53–––25.43	6.11–––21.80	
median (Q1, Q3)	14.26 (11.32, 16.94)	13.85 (11.11, 16.14)	

Dimensional Card Change Sort, age corrected standard score			0.020
No. (%)	47 (100%)	75 (100%)	
mean (SD)	94.14 (16.26)	101.09 (15.53)	
range	66.49–––148.00	71.00–––145.00	
median (Q1, Q3)	95.00 (81.54, 103.02)	100.83 (89.19, 111.73)	

List Sorting Working Memory, age corrected standard score			0.294
No. (%)	46 (97.87%)	75 (100%)	
mean (SD)	102.61 (14.83)	105.48 (14.37)	
range	66.93–––135.00	80.00–––147.00	
median (Q1, Q3)	101.90 (94.47, 110.05)	105.67 (97.43, 111.83)	

Flanker Attention & Inhibitory Control, age corrected standard score			0.056
No. (%)	47 (100%)	75 (100%)	
mean (SD)	95.57 (14.13)	100.56 (13.69)	
range	66.00–––128.00	67.00–––138.00	
median (Q1, Q3)	95.99 (84.78, 103.41)	101.73 (90.22, 109.52)	

Fluid Cognition Composite, age corrected standard score			0.027
No. (%)	45 (95.74%)	75 (100%)	
mean (SD)	96.85 (18.05)	105.39 (21.41)	
range	68.00–––133.20	57.23–––145.94	
median (Q1, Q3)	94.5 (80.23, 110.00)	107.05 (89.98, 120.99)	

Crystallized Cognition Composite, age corrected standard score			< 0.001
No. (%)	45 (95.74%)	74 (98.67%)	
mean (SD)	105.75 (13.33)	115.66 (15.53)	
range	78.00–––143.00	85.43–––146.00	
median (Q1, Q3)	105.35 (99.53, 111.97)	114.06 (104.45, 129.27)	

WISC-IV* Age at Completion, y			0.409
No. (%)	30 (63.83%)	53 (70.67%)	
mean (SD)	12.87 (2.93)	12.32 (2.86)	
range	6.53–––16.95	6.11–––16.97	
median (Q1, Q3)	12.64 (11.10, 15.21)	12.67 (10.45, 14.63)	

Digit Span, scaled score			0.063
No. (%)	30 (63.83%)	53 (70.67%)	
mean (SD)	9.23 (3.02)	10.47 (2.78)	
range	4.00–––16.00	4.00–––18.00	
median (Q1, Q3)	9.50 (7.00, 11.00)	11.00 (9.00, 12.00)	

**BRIEF-2 Parent Report** Age at Completion, y			0.456
No. (%)	30 (63.83%)	54 (72.00%)	
mean (SD)	12.87 (2.93)	12.38 (2.86)	
range	6.53–––16.95	6.11–––16.97	
median (Q1, Q3)	12.64 (11.10, 15.21)	12.70 (10.52, 14.75)	

Shift, T-score			0.107
No. (%)	30 (63.83%)	54 (72.00%)	
mean (SD)	49.57 (12.25)	45.46 (8.04)	
range	36.00–––88.00	36.00–––66.00	
median (Q1, Q3)	46.00 (40.00, 56.00)	42.50 (38.00, 51.75)	

Working Memory, T-score			0.029
No. (%)	30 (63.83%)	54 (72.00%)	
mean (SD)	55.53 (13.32)	49.28 (9.71)	
range	35.00–––82.00	36.00–––78.00	
median (Q1, Q3)	56.00 (42.00, 66.00)	47.00 (42.25, 54.50)	

Inhibit, T-score			0.132
No. (%)	30 (63.83%)	54 (72.00%)	
mean (SD)	48.70 (7.61)	46.31 (6.47)	
range	36.00–––74.00	37.00–––66.00	
median (Q1, Q3)	48.00 (44.25, 51.75)	44.00 (42.00, 50.25)	

Notes. * = 2 subjects assessed with WISC-V. WISC-IV = Wechsler Intelligence Scale for Children Fourth Edition. BRIEF-2 = Behavior Rating Inventory of Executive Function Second Edition.

For transparency, full regression outputs for all analyses conducted—including all regions, metrics, and cognitive measures—are available in the Supplement.

### 3.2 Global network organization does not differ by CHD status

Prior to binarization, we saw no significant difference in the average functional connectivity values between CHD and control groups. After adjusting for age and sex, we observed no significant differences in global network connectivity based on metrics such as global efficiency, assortativity, density, transitivity, small-world coefficient, or the number of components across all thresholds and weighted networks. Furthermore, these metrics did not yield a significant coefficient in the interaction term when fitting to the neuropsychological measures.

In both cohorts, networks exhibited assortative behavior and demonstrated small-world characteristics. However, it is noteworthy that the small-world coefficient exhibited a decreasing trend as the network became denser with lighter thresholds (Supplemental Fig. 2).

### 3.3 Local network associations with executive function measures

**Fig. 3:**
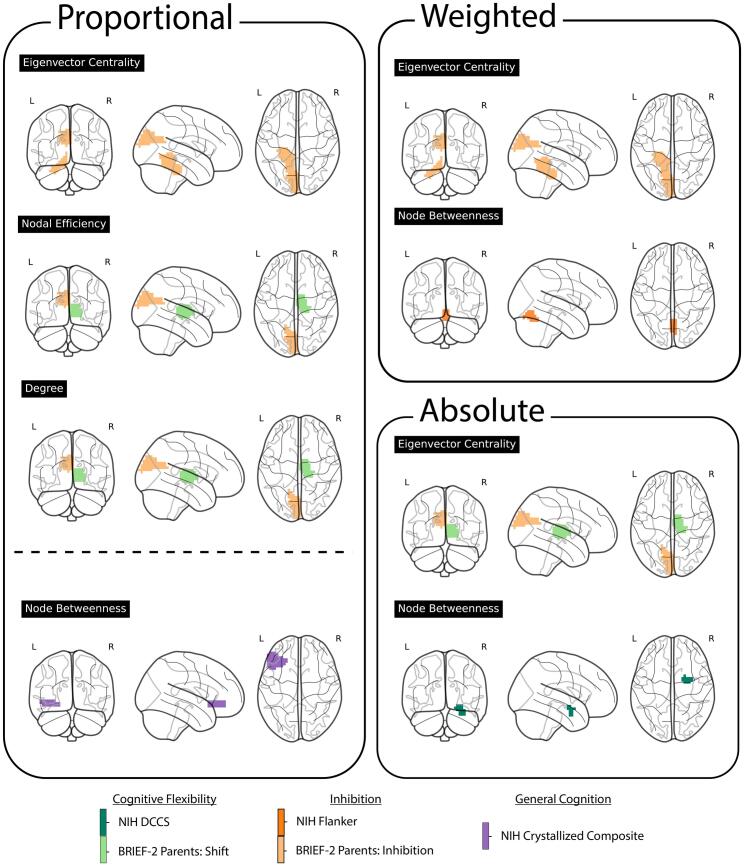
Neuropsychological Measures and Brain Regions. Fig. 3 identifies the brain regions in which the interaction between CHD and network metrics reached statistical significance, along with the cognitive measures associated with each region. Notably, eigenvector centrality in temporal brain regions showed fluctuations with the WISC-IV Digit Span across two methods. Furthermore, discrepancies in eigenvector centrality in the right Lobule IV and V of the cerebellar hemisphere were observed with the BRIEF-2 Inhibition score across two methods. The right thalamus displayed differences with the BRIEF-2 Shift across three graph measures and two methods. Similarly, the Cuneus exhibited variations with the BRIEF-2 Shift across three graph measures, particularly with eigenvector centrality showing inconsistencies across all three methods. These results underscore patient-specific differences in graph measures across temporal, subcortical, and occipital brain regions across various cognitive assessments. Below the dotted line are results from NIHTB Crystalized Composite.

The detected differences in our assessment of cognitive outcomes were predominantly in local measures of specific brain regions rather than in global measures across the entire brain network. The outcomes of the regression analyses using weighted metrics are shown in Table 3, absolute metrics are presented in Table 4, and proportional metrics in Table 5. Fig. 3 shows regions exhibiting significant interaction terms (i.e., differing relationships between graph measures and cognitive scores in the presence of CHD) and the corresponding cognitive measures.

**Table 3:** Regional Weighted Network Correlations with Neuropsychological Testing. Note: Higher scores generally indicate better performance, except on the BRIEF-2, where higher scores correspond to poorer functioning. Accordingly, a positive interaction coefficient for the BRIEF-2 reflects poorer functioning with increasing graph metric values, whereas a positive coefficient for all other measures reflects improved performance.

Domain	NeuroCog	Region	Measure	Graph Coeff	Graph P	CHD Coeff	CHD p	Interaction Coeff	Interaction p
EF − Inhibition	BRIEF-2 Parent Inhibit	Left Cuneus	Eigenvector Centrality	−2.76	0.0021	3.46	0.0203	5.98	0.00015
EF − Inhibition	BRIEF-2 Parent Inhibit	Left Lobule IV, V of cerebellar hemisphere	Eigenvector Centrality	−2.06	0.0120	3.02	0.0411	5.63	0.00012
EF − Inhibition	NIH Flanker Inhibitory Control and Attention Test	Lobule VI of vermis	Node Betweenness	2.63	0.0487	−5.68	0.0265	−10.74	0.00079

**Table 4:** Absolute Thresholding Correlations with Neuropsychological Testing. Note: Higher scores generally indicate better performance, except on the BRIEF-2, where higher scores correspond to poorer functioning. Accordingly, a positive interaction coefficient for the BRIEF-2 reflects poorer functioning with increasing graph metric values, whereas a positive coefficient for all other measures reflects improved performance.

Domain	NeuroCog	Region	Measure	Graph Coeff	Graph P	CHD Coeff	CHD p	Interaction Coeff	Interaction p
EF − Cognitive Flexibility	BRIEF-2 Parent Shift	Right Thalamus	Eigenvector Centrality	1.43	0.1986	6.45	0.0049	−9.06	0.00081
EF − Cognitive Flexibility	NIH DCCS	Right Amygdala	Node Betweenness	4.56	0.0284	−7.38	0.0107	−10.84	0.00073
EF − Inhibition	BRIEF-2 Parent Inhibit	Left Cuneus	Eigenvector Centrality	−3.16	0.0011	3.43	0.0216	5.92	0.00022

**Table 5:** Proportional Thresholding Correlations with Neuropsychological Testing. Note: Higher scores generally indicate better performance, except on the BRIEF-2, where higher scores correspond to poorer functioning. Accordingly, a positive interaction coefficient for the BRIEF-2 reflects poorer functioning with increasing graph metric values, whereas a positive coefficient for all other measures reflects improved performance.

Domain	NeuroCog	Region	Measure	Graph Coeff	Graph P	CHD Coeff	CHD p	Interaction Coeff	Interaction p
EF − Cognitive Flexibility	BRIEF-2 Parent Shift	Right Thalamus	Nodal Efficiency	1.33	0.2167	7.10	0.0028	−10.43	0.00069
EF − Cognitive Flexibility	BRIEF-2 Parent Shift	Right Thalamus	Degree	1.37	0.2282	6.96	0.0033	−9.87	0.00072
EF − Inhibition	BRIEF-2 Parent Inhibit	Left Cuneus	Nodal Efficiency	−2.94	0.0049	3.16	0.0357	5.59	0.00042
EF − Inhibition	BRIEF-2 Parent Inhibit	Left Cuneus	Degree	−2.92	0.0042	3.17	0.0347	5.57	0.00043
EF − Inhibition	BRIEF-2 Parent Inhibit	Left Cuneus	Eigenvector Centrality	−2.53	0.0052	3.41	0.0238	5.74	0.00042
EF − Inhibition	BRIEF-2 Parent Inhibit	Left Lobule IV, V of cerebellar hemisphere	Eigenvector Centrality	−2.23	0.0073	3.02	0.0411	5.67	0.00014
General Cognition	NIH Crystallized Composite	Left Inferior frontal, pars orbitalis	Node Betweenness	−9.70	0.0070	−10.99	0.0001	15.72	0.00029

No significant results were found in objectively measured or parent-rated working memory, including the WISC-IV/V Digit Span, NIHTB LSWM, or BRIEF-2 Working Memory. These null findings indicate that alterations in local network properties did not correspond to differences in working memory performance in this cohort.

In contrast, several regions demonstrated significant associations with parent-rated inhibitory control and mental flexibility. For the BRIEF-2 Inhibit scale, eigenvector centrality in the left cerebellar Lobule IV–V showed a significant interaction effect across weighted and proportional analyses. For the BRIEF-2 Shift scale, the right thalamus exhibited significant interaction effects in degree, nodal efficiency, and eigenvector centrality across proportional and absolute thresholds. The cuneus showed consistent associations across degree, nodal efficiency, and eigenvector centrality, with eigenvector centrality significant across all three approaches. Together, these findings highlight cerebellar, thalamic, and occipital regions as key areas where local network organization relates differently to executive functioning in CHD.

### 3.4 Local network associations with general cognitive measures

Whereas Section 3.3 focuses on executive function outcomes, this section examines associations between local network metrics and broader general cognitive ability. In contrast to findings for executive functions, the general cognition scores (e.g., Crystallized Composite and Fluid Composite) yielded fewer significant results. We observed no significant associations with Fluid Composite Scores. For Crystallized Composite Score, there was a significant positive association with node betweenness in the left inferior frontal gyrus pars opercularis—a region in the frontal lobe. This was the only result observed among general cognition scores and was seen in proportionally binarized networks.

### 3.5 Spatial patterns of Network–Cognition interaction effects

**Fig. 4:**
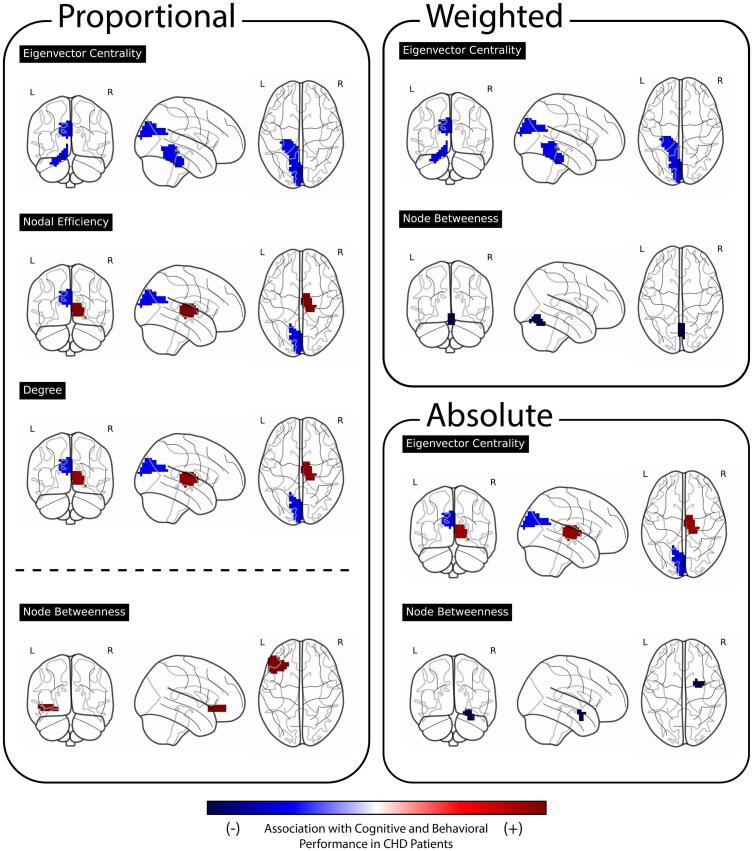
Regional associations with executive function and cognitive performance. Red indicates associations reflecting better cognitive performance and blue indicates associations reflecting worse performance. For visualization, associations involving BRIEF-2 scores (where higher values indicate poorer functioning) were direction-corrected so that the sign aligns with the interpretation used for all other cognitive measures. Temporal lobe regions generally display a positive correlation with cognitive performance, while occipital regions exhibit negative associations. Subcortical regions present a mix of findings, as illustrated by the amygdala's association with decreased performance when considering the interaction between CHD and node betweenness. Conversely, the right thalamus shows increased performance linked to interactions between CHD and degree, nodal efficiency, and eigenvector centrality. Below the dotted line are results from NIHTB Crystalized Composite. (For interpretation of the references to colour in this figure legend, the reader is referred to the web version of this article.)

Fig. 3 displays the regions showing statistically significant interaction effects and the cognitive measures they are associated with. In contrast, Fig. 4 illustrates the direction of these effects across the brain, indicating whether increasing network metric values relate to better or worse cognitive performance in CHD. Because Fig. 4 adjusts for score directionality, including the reversed interpretation of BRIEF scores, it reflects improvement or decline in cognition rather than changes in raw scores.

Fig. 4 shows the spatial distribution of correlations between each region’s network metric and its predicted effect on cognitive performance in the presence of CHD (i.e., the regression interaction term). Regions in the cerebellum and occipital lobe generally exhibited negative correlations, suggesting poorer performance with increasing network metric values in CHD.

Subcortical regions showed mixed patterns depending on the metric. For example, the interaction between CHD and node betweenness in the amygdala was associated with poorer performance on the NIHTB Dimensional Change Card Sort (DCCS), a task assessing mental flexibility. In contrast, the interaction of CHD with degree, nodal efficiency, and eigenvector centrality in the right thalamus were associated with *improved* parent-rated mental flexibility on the BRIEF-2 Shift.

### 3.6 Ad-Hoc sub-analysis for age groups

Overall, the associations observed in the age-stratified analyses did not replicate consistently within the full dataset. These inconsistencies suggest that some relationships identified in the full sample may reflect sample heterogeneity, residual age effects, or potential spurious findings. We emphasize that the analyses in this manuscript were not corrected for multiple comparisons, which is an important limitation.

Because the outcome measures are age-corrected standard scores, developmental effects on test performance are already accounted for at the instrument level. Thus, the regression models are not attempting to “control away” raw developmental change but instead evaluate variance that remains after age adjustment. Age stratification further reduces sample size within each subgroup, decreasing statistical power and increasing the likelihood of unstable estimates. The inconsistent findings between the 8–11 and 12 + groups may therefore reflect reduced power rather than true developmental differences.

Results from the age-stratified sub-analysis are presented in Supplemental Table 3. These findings underscore the need for larger, age-balanced samples in future developmental neuroimaging studies, a point further emphasized in the manuscript’s limitations section.

### 3.7 Ad-Hoc sub-analysis for system segregation

System segregation within the Default Mode Network (DMN) did not differ significantly between CHD and control participants. In the regression model including age and sex as covariates, the CHD coefficient was negative (β = –0.041) but not statistically significant (p = 0.1345), indicating no evidence of reduced or increased DMN segregation associated with CHD status in this sample. These findings suggest that large-scale functional differentiation of the DMN, as measured by system segregation, is not markedly altered in CHD within the limits of our dataset.

## 4 Discussion

### 4.1 Overview of findings

The current study explored differences in brain connectivity between CHD patients and a healthy sample and associated links with executive functioning to better understand functional reorganization associated with CHD. Recognizing the challenges in network and threshold selection, we used both weighted and binarized networks. Overall, our findings revealed patient differences among graph measures in cerebellar, subcortical, and occipital brain regions across several measures of executive functioning. Comparatively, fewer associations emerged with broader measures of cognition that were explored as a comparison, and no significant findings were seen with a broader indicator of fluid cognition. In CHD patients, node betweenness in the left inferior frontal gyrus, pars orbitalis, was positively correlated with crystalized cognition, even though both increased node betweenness in this region and the presence of CHD were individually negatively correlated with crystalized cognition.

### 4.2 Methodological Considerations: Weighted vs. Thresholded networks

The use of graph metrics on networks with absolute thresholds emphasizes edge value differences that may be the result of variations in connectivity strength. Proportional metrics, on the other hand, allow for the control of the number of edges while selecting the strongest edges without selecting a fixed edge value, facilitating an investigation specifically into differences in the topology and distribution of edges. The weighted metrics offer a balanced approach, accounting for both the number and strength of edges, albeit with the acknowledgment that this method may be more susceptible to noise. Our study saw repeated results across methods. For instance, all three methods revealed differences in the interaction of CHD with network metrics in the left cuneus region of the brain, indicating variations in both the strength and topology of edges adjacent to this region. While the cerebellar regions exhibited differences in both weighted and proportional results, they showed no detectable differences when using absolute thresholds. This suggests that, while the topology of the cerebellum's strongest edges may differ, the overall strength of the edges remains consistent. Alternatively, subcortical regions displayed differences in proportional and absolute thresholded networks, but not in weighted networks. This may imply that there are variations in both regional edge strength and topology among the strongest edges in the network but not necessarily all edges.

### 4.3 Regional network alterations and executive function outcomes

Regression analyses using graph metrics on networks constructed with weighted, absolute, and proportionally thresholded networks highlighted localized discrepancies in neuropsychological outcomes, particularly within specific brain regions, rather than global measures. Notably, differences were observed in the presence of CHD in regions such as the occipital, and subcortical areas, with varied associations between graph measures and cognitive scores. For instance, interactions between CHD and eigenvector centrality in the left cuneus is associated with poorer parent-reported mental flexibility, whereas interactions between CHD and eigenvector centrality in the right thalamus predicted improved set shifting, suggesting nuanced impacts on brain function. These results underscore the complex interplay between brain network metrics, cognitive performance, and the presence of CHD, with dynamic, interacting compensatory mechanisms. Observing more local discrepancies may underscore unique neurological pathways and compensatory mechanisms underlying cognitive skills. For example, the cuneus and the thalamus have been previously associated with measures of mental flexibility (Kim et al., 2011). However, this pattern may also reflect a broader characteristic of the neurological development among those with CHD, which could exhibit more localized rather than global differences, particularly in functional networks. Further investigation is needed to disentangle whether these findings are primarily driven by the cognitive domain examined or by inherent network-level differences in CHD.

### 4.4 Specific regional Mechanisms: Occipital, Amygdalar, and thalamic contributions

Building on these region-specific differences, CHD-related disruptions in brain connectivity may have functional consequences for executive abilities, particularly inhibitory control. In the occipital regions, such as the cuneus, alterations in eigenvector centrality were observed to have implications for inhibitory control. Decreases in eigenvector centrality within the cuneus were associated with diminished inhibitory performance. These findings suggest that more centrally accessible occipital network connectivity may contribute to poorer inhibition among CHD patients. While the occipital lobe is primarily associated with visual processing, emerging research suggests it may also play a role in executive function deficits, particularly when its connectivity with other brain regions is disrupted (Park et al., 2011, Santangelo et al., 2017, Traianou et al., 2019).

Beyond the occipital lobe, subcortical regions also demonstrate altered connectivity patterns in CHD, further highlighting the role of network centrality in executive function outcomes. Increases in node betweenness in the amygdala exhibit a large negative correlation with mental flexibility as indexed with the NIHTB DCCS, suggesting that the amygdala taking a more central role in connecting other brain regions in CHD patients is associated with poorer executive function. The thalamus, another subcortical region, sees a positive relationship between centrality and outcome. The difference in how the amygdala and thalamus relate to cognitive performance may be linked to the specific centrality measures that show changes in each region. Eigenvector centrality, which is altered in the thalamus, reflects the influence of a region based on its connections to other highly connected regions, suggesting that the thalamus may support cognitive function through numerous strong ties within the brain network. This interpretation aligns with the hypothesis that the thalamus acts as a critical hub involved in integrating information across cortical networks (Hwang et al., 2017). In contrast, betweenness centrality, which is affected in the amygdala, measures how often a region acts as a bridge between other nodes rather than the number of neighbors it has. This may indicate that when the amygdala plays a more central bridging role, it is associated with lower executive function performance. Prior studies have similarly observed that altered functional activity in the amygdala is linked to poorer behavioral outcomes (Fan et al., 2021, Jacob et al., 2022). Moreover, research supports the role of both the thalamus and amygdala as network hubs (Jacob et al., 2022, Santos et al., 2023). Another study also identified distinct relationships between thalamic and amygdala functional connectivity in autism spectrum disorder, underscoring their differing contributions to brain network organization (Iidaka et al., 2019). Altogether, our findings align with prior studies, and the observation that most group differences were found in centrality measures suggests a potential reorganization of brain networks.

### 4.5 Cerebellar connectivity and cognitive outcomes

In addition to the findings in subcortical regions, recent studies have highlighted alterations in cerebellar function among CHD patients, further complicating the relationship between brain network connectivity and cognitive performance. Previous research has observed alterations in cerebellar function among patients with CHD. A separate study used the NIHTB with the same set of patients observed altered cerebello-cerebral connectivity and increased fractional anisotropy correlated with poorer cognitive functioning (Sahel et al., 2023). Another study also delved into the cerebellum of CHD patients and observed associations between cerebellar volume and cognitive test scores (Badaly et al., 2022). These findings align with prior hypotheses that cerebro-cerebellar circuits play an important role in skill acquisition and early disruption to its development could result in long term alterations to its neural circuits impacting cognition and behavior (Cook et al., 2023, Stoodley, 2016).

### 4.6 Evidence for compensatory or hyperconnectivity mechanisms

However, not all CHD-related network alterations are linked to poorer outcomes; in some cases, increased centrality in key regions appears to correlate with better cognitive performance. Some measured interactions between CHD and key regional network metrics appear to be correlated with better cognitive performance scores in measures with no difference in executive functioning between groups. For example, the interaction of CHD and a more central and efficient right thalamus appears to correlate with improved cognitive flexibility, as assessed by the BRIEF-2 Shift. Taken together, these observations could be explained by a potential compensatory or hyperconnectivity response in these brain regions (Hillary & Grafman, 2017).

### 4.7 Relationship to structural network findings in CHD

We have previously investigated structural network analyses in CHD patients and their association with cognitive performance, yielding a mix of findings. Panigrahy et al. (2015) utilized diffusion tensor imaging (DTI), graph analysis, and statistical mediation models to identify neurocognitive impairments in adolescents who underwent early infancy repair for dextro-transposition of the great arteries (d-TGA), linking these impairments to variations in the overall topology of the white matter structural network (Panigrahy et al., 2015). Similarly, Schmithorst et al. (2016) discovered differences at both global and regional levels, highlighting the role of global white matter structural network topology in mediating adverse ADHD outcomes among adolescents with d-TGA (Schmithorst et al., 2016). While prior studies indicate structural brain network reorganization, our findings suggest that the global functional network remains largely undifferentiated, with predominantly regional differences. Thus, functional network analysis may instead reflect regional, rather than global, compensatory mechanisms contributing to both higher variance but still overall normative performance in broad cognitive tests observed in CHD patient populations (Badaly et al., 2022, Wallace et al., 2023).

### 4.8 Convergence with prior functional connectivity studies

Other functional connectivity analyses across different imaging modalities and age groups in CHD patients have shown similar patterns. Studies using EEG and rsfMRI data have reported preserved global network metrics but regional disturbances, particularly in subcortical areas and the brainstem (De Asis-Cruz et al., 2023, De Asis-Cruz et al., 2018, Wehrle et al., 2023). Functional near-infrared spectroscopy (fNIRS) studies in CHD infants also found no global differences but identified altered relationships between network metrics and language/communication skills (Provost et al., 2024, Provost et al., 2023). Collectively, these findings reinforce the growing evidence that CHD primarily affects regional rather than global functional networks across the lifespan.

### 4.9 Limitations

This study has limitations. Firstly, a common limitation in most functional connectivity studies on CHD patients is small sample size. In fact, our study N = 122 is still one of the larger datasets compared to previously published work: N = 38 (Wehrle et al., 2023), N = 82 (Enguix et al., 2022), N = 112 (De Asis-Cruz et al., 2018), N = 182 (De Asis-Cruz et al., 2023), N = 51 (Provost et al., 2023) N = 42 (Provost et al., 2024). However, given the large number of features and outcomes observed, some of our findings may be spurious. Similarly, some of our findings do not adhere to the underlying assumptions of related outcome measures. For example, we see structural overlap across Inhibition measures, namely Node Betweenness is associated with NIH Flanker, and Eigenvector Centrality is associated with the BRIEF-2 Parent Inhibition, but did not observe the same graph metrics associated with the same cognitive domain. Thus, these findings may be spurious or associated with an unmeasured latent variable such as region-specific vascular dysfunction or structural abnormality (Jain et al., 2014, Schmithorst et al., 2022a, Schmithorst et al., 2022b). Future work with multicenter designs and developing larger datasets can help mitigate sample size issues by generating harmonized datasets, or by independently replicating our findings to allow for a more robust multi-comparison statistical approach.

As is often a challenge in imaging studies, our demographic tends to be biased towards high- or normative-performing participants, and neurocognitive results can sometimes be skewed towards a normative ceiling with a longer tail of poor performing individuals. We observe this in the BRIEF-2 results. For all BRIEF-2 clinical scales and indexes, T scores from 60 to 64 are considered mildly elevated, and T scores from 65 to 69 are considered potentially clinically elevated. T scores at or above 70 are considered clinically elevated. While this is a limitation, the effect size we do see between CHD and controls is still observable despite a bias towards high-performing participants.

Additionally, we did not include social determinants of health, such as socioeconomic status which are known to influence cognitive development, and particularly those with CHD (Bucholz et al., 2021, Lee et al., 2024). In Table 1, while our study observed differences in maternal education, we did not see a difference in Child Opportunity Index (COI) which assesses neighborhood-level socio-economic factors rather than individual socio-economic factors. Future works should further investigate the effects of sociodemographic factors on brain network development while also using large, diverse datasets. We limited our current analyses to executive functioning, mindful that increased analyses can lead to increased spurious findings and because of the important role that executive functioning plays in development and quality of life. However, additional attention should be directed toward the range of cognitive domains within larger studies. Finally, this study primarily reports on the NIH Toolbox, which has been previously used to identify regional brain differences in CHD and to understand the cognitive and psychosocial functioning of those with CHD (Badaly et al., 2022, Calderon et al., 2019, Cousino et al., Sahel et al., 2023, Siciliano et al., 2021, Wallace et al., 2023), and should be further evaluated on clinically-validated neurocognitive batteries.

## 5 Conclusion

Our exploratory study investigated the relationship between CHD, functional brain network organization, and cognitive performance with a focus on executive functioning in children, adolescents, and young adults. Utilizing regression analyses on graph metrics derived from weighted and binarized networks, we identified localized associations between specific brain regions and executive function outcomes. Notably, interactions between CHD and regional network metrics demonstrated nuanced impacts on cognitive function, highlighting potential compensatory mechanisms involving key brain regions. Our findings underscore the importance of considering both global and regional network characteristics in understanding cognitive deficits associated with CHD, and identifying robust signals across methodology and threshold selection. Moreover, our study contributes to the growing body of literature exploring cognitive impairments in CHD patients. Further research employing multimodal approaches leveraging both functional and structural imaging may provide deeper insights into the biological underpinnings of cognition in those with CHD.

## 6 Data and code availability

The code used in this study is openly available and can be accessed at the following GitHub repository: https://github.com/PIRCImagingTools/Sim_Funcky_Pipeline.

## CRediT authorship contribution statement

**Joy Roy:** Writing – review & editing, Writing – original draft, Visualization, Software, Methodology, Investigation, Formal analysis, Conceptualization. **William Reynolds:** Writing – review & editing, Methodology. **Julia Wallace:** Writing – review & editing, Visualization. **Daryaneh Badaly:** Writing – review & editing, Supervision. **Rafael Ceschin:** Writing – review & editing, Supervision, Project administration, Methodology, Funding acquisition, Conceptualization.

## Funding

This work was supported by the Department of Defense (W81XWH-16–1-0613), the National Heart, Lung, and Blood Institute (R01 HL152740-1, R01 HL128818-05), and the National Heart, Lung and Blood Institute with National Institute on Aging (R01HL128818-05 S1). Its contents are solely the responsibility of the authors and do not necessarily represent the official views of the NIH. We also acknowledge Additional Ventures for support and National Library of Medicine (5 T15-LM007059-3 [to J.R. and W.R]).

## Declaration of competing interest

The authors declare that they have no known competing financial interests or personal relationships that could have appeared to influence the work reported in this paper.

## Data Availability

Data will be made available on request.
