# Supplementary material for: Functional network organization is locally atypical in children, adolescents, and young adults with congenital heart disease
Source: Neuroimage Clin. 2026 Feb 13;49:103965. doi: 10.1016/j.nicl.2026.103965 (PMC12926604; doi:10.1016/j.nicl.2026.103965)
Supplement: Supplementary Data 1 [file mmc1.docx]

**Supplement**


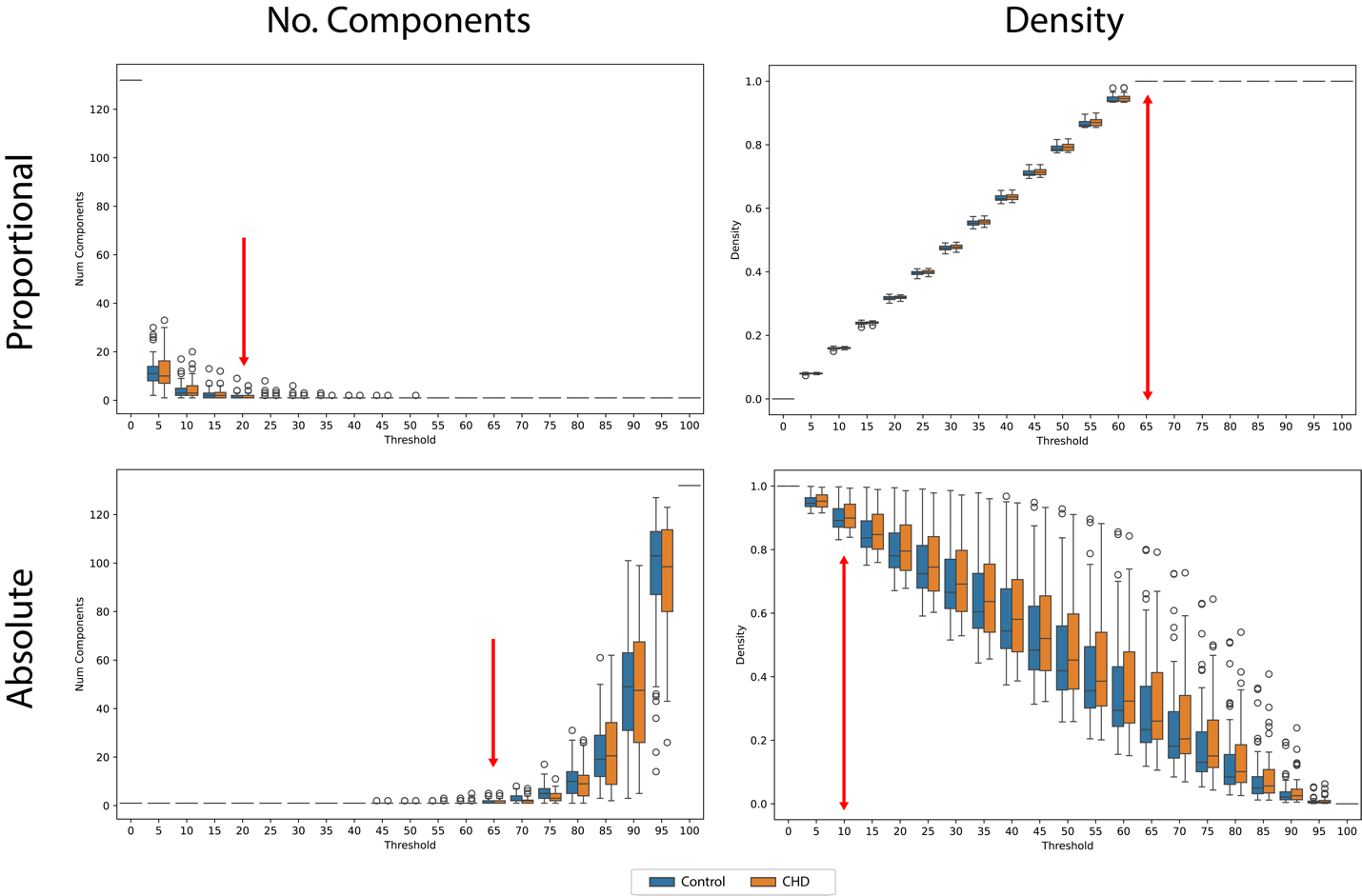


Supplemental Figure 1. Absolute thresholding had a lower threshold of 0.10 to mitigate spurious connections and an upper threshold of 0.65 to limit the number of components in the network, an anatomical constraint. Proportional thresholding ranged from 0.20 to limit the number of components to 0.65, where all networks became fully connected. Note: A proportional threshold takes a top down approach where it selects the strongest X% of edges first, whereas an absolute thresholds take a bottom up approach keeping edges with strength >= X.


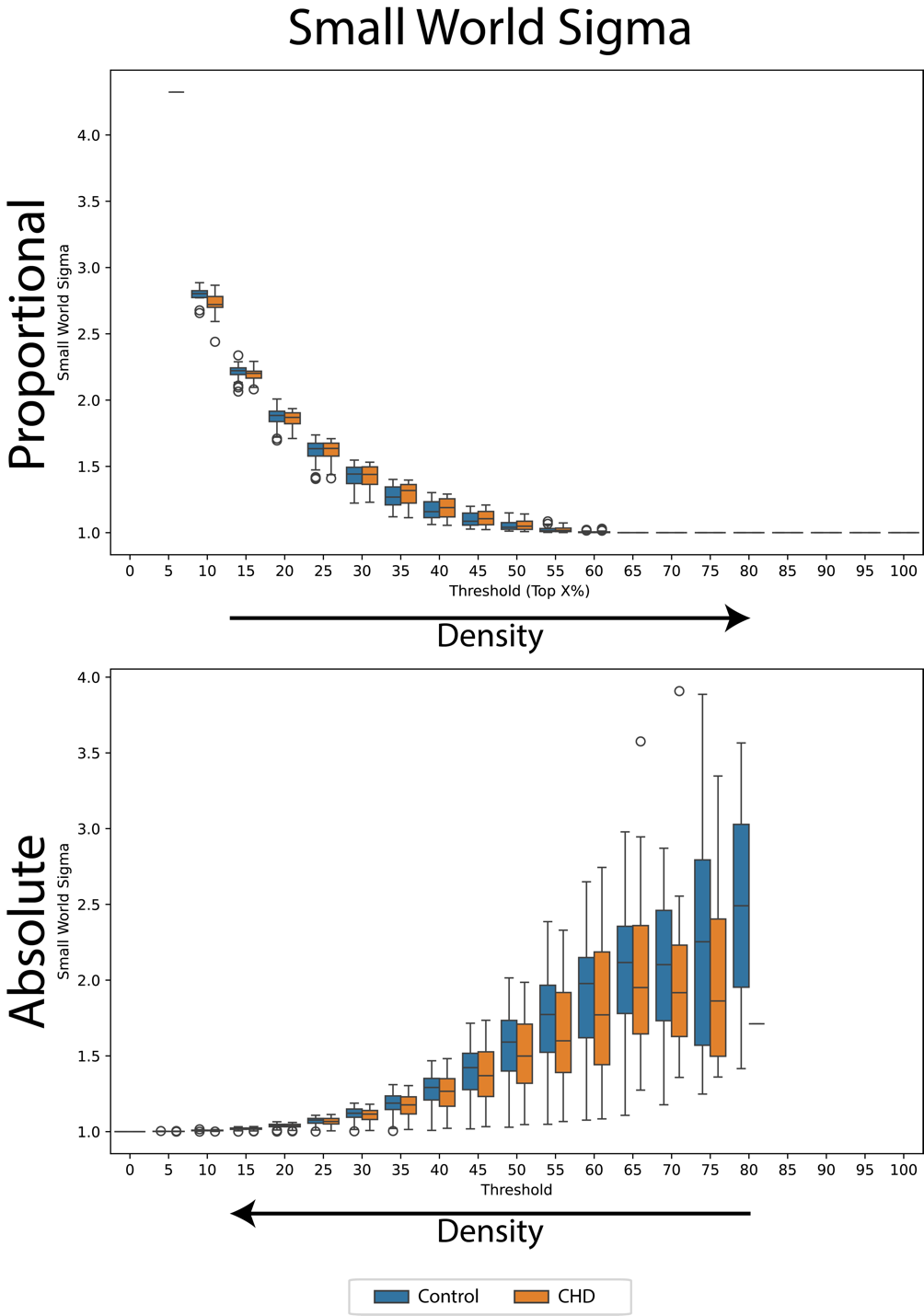


Supplemental Figure 2. The small World Coefficient (sigma) tends to be larger in sparse graphs and decreases as thresholds become more lenient, resulting in denser networks. The Small World Coefficient was calculated only in networks with a single component. The notable difference in variance between absolute and proportional thresholding can be attributed to the fact that proportional thresholding controls for network density, whereas absolute thresholding does not, leading to highly variable edge counts in patient networks even at a single threshold.


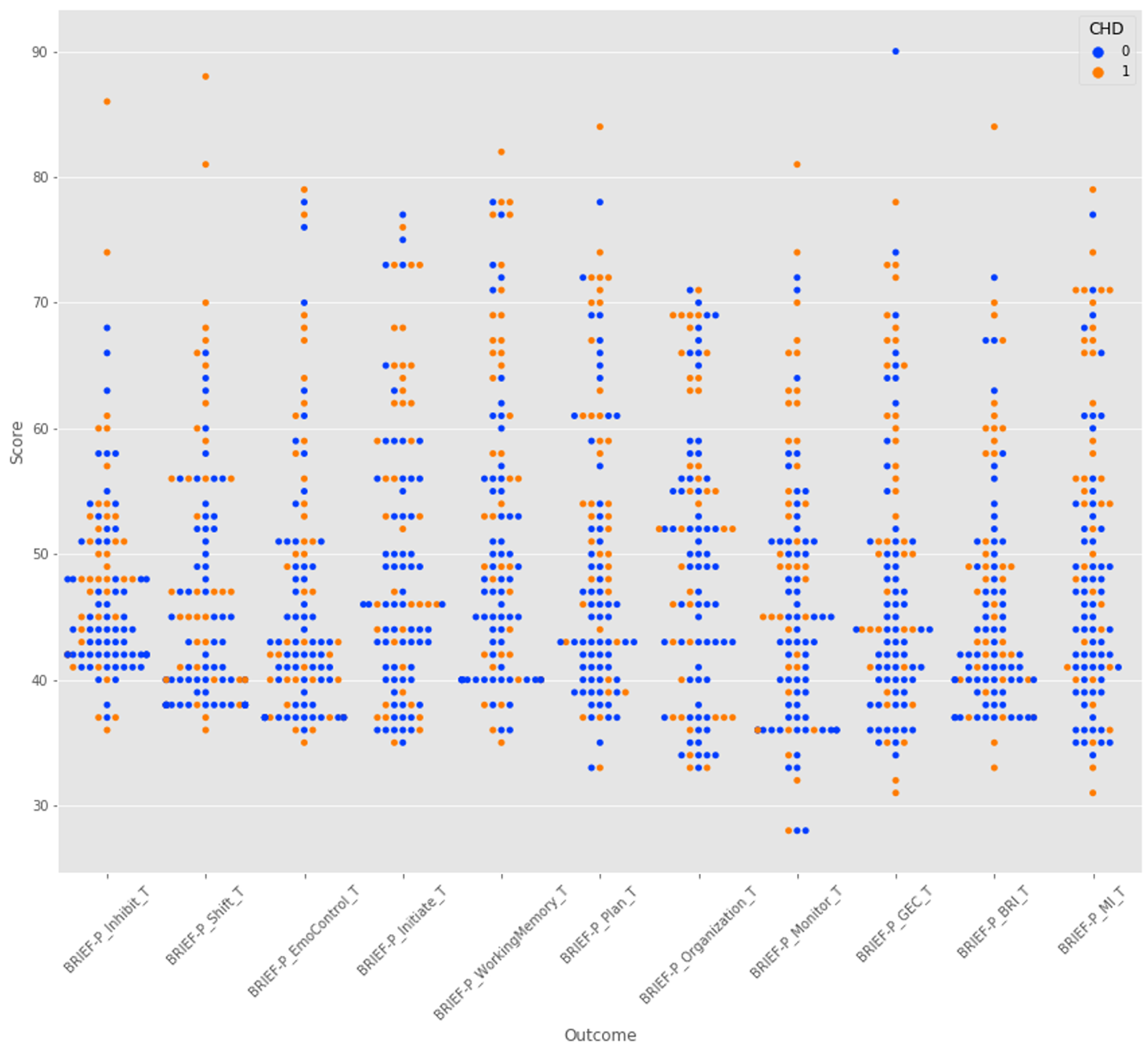


*Supplemental Figure 3. Distribution of BRIEF-2 T-scores across all collected subscales for CHD and control participants. Only a subset of these measures was used in the present study; the full set is shown here to illustrate overall score distributions. Scores show the expected clustering within the normative range with a longer tail of elevated values. Reference ranges (mildly elevated: 60–64; potentially clinically elevated: 65–69; clinically elevated: ≥70) are provided for context.*

*Supplemental Table 1. Characteristics of Cardiac Lesions in CHD Patients*


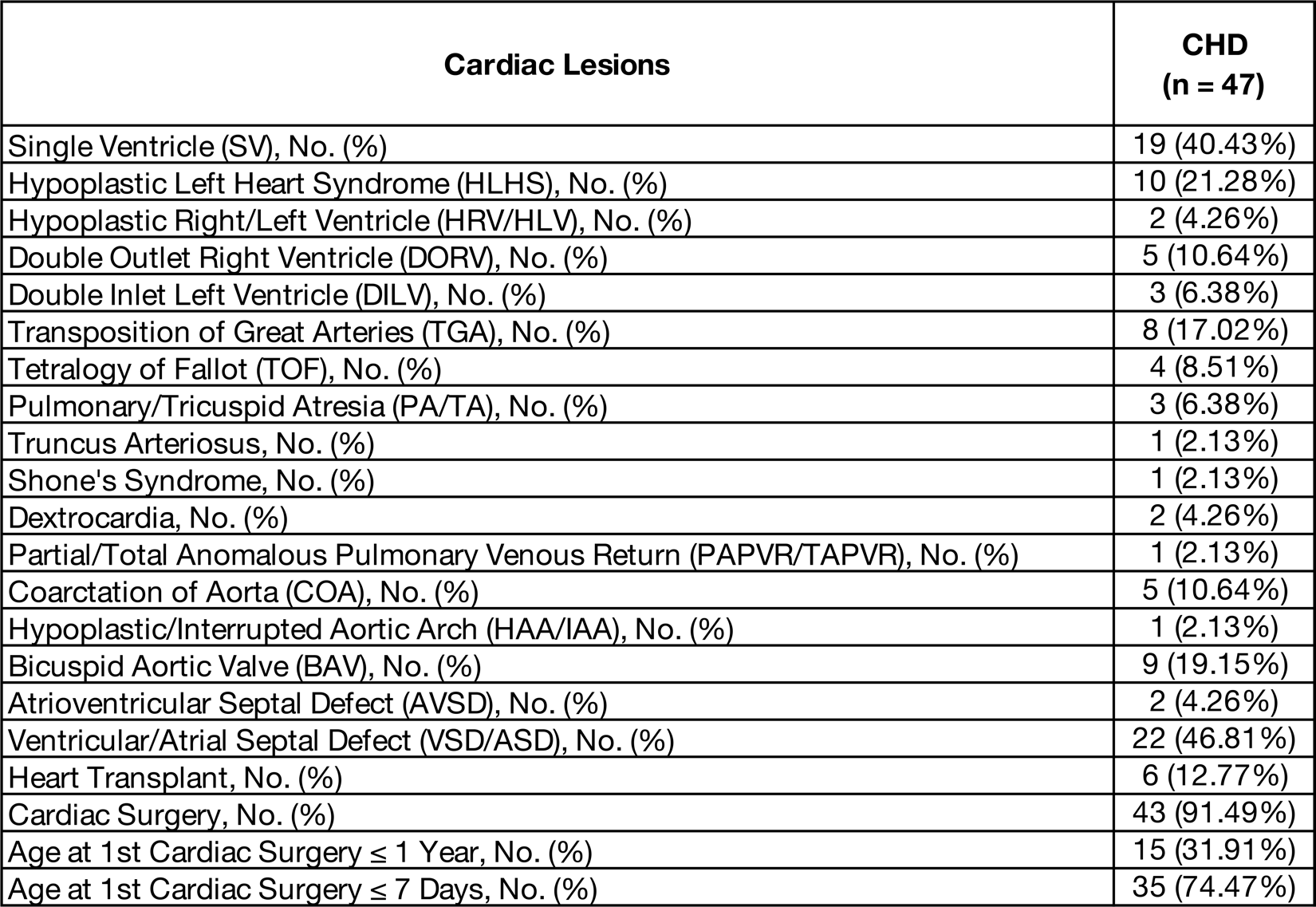


Supplemental Table 2. Sample Sizes for Ad-Hoc Sub-analysis for Age Groups. *Note: due to the low sample size for the 0 to 7 group, it was not included in the stratification analysis.*

| **Group** | **N Control** | **N CHD** | **N Total** |
| --- | --- | --- | --- |
| 0 to 7 | 6 | 2 | 8 |
| 8 to 11 | 19 | 15 | 34 |
| 12 plus | 50 | 30 | 80 |

Supplemental Table 3. Regression Analysis for Ad-Hoc Sub-analysis for Age Groups.


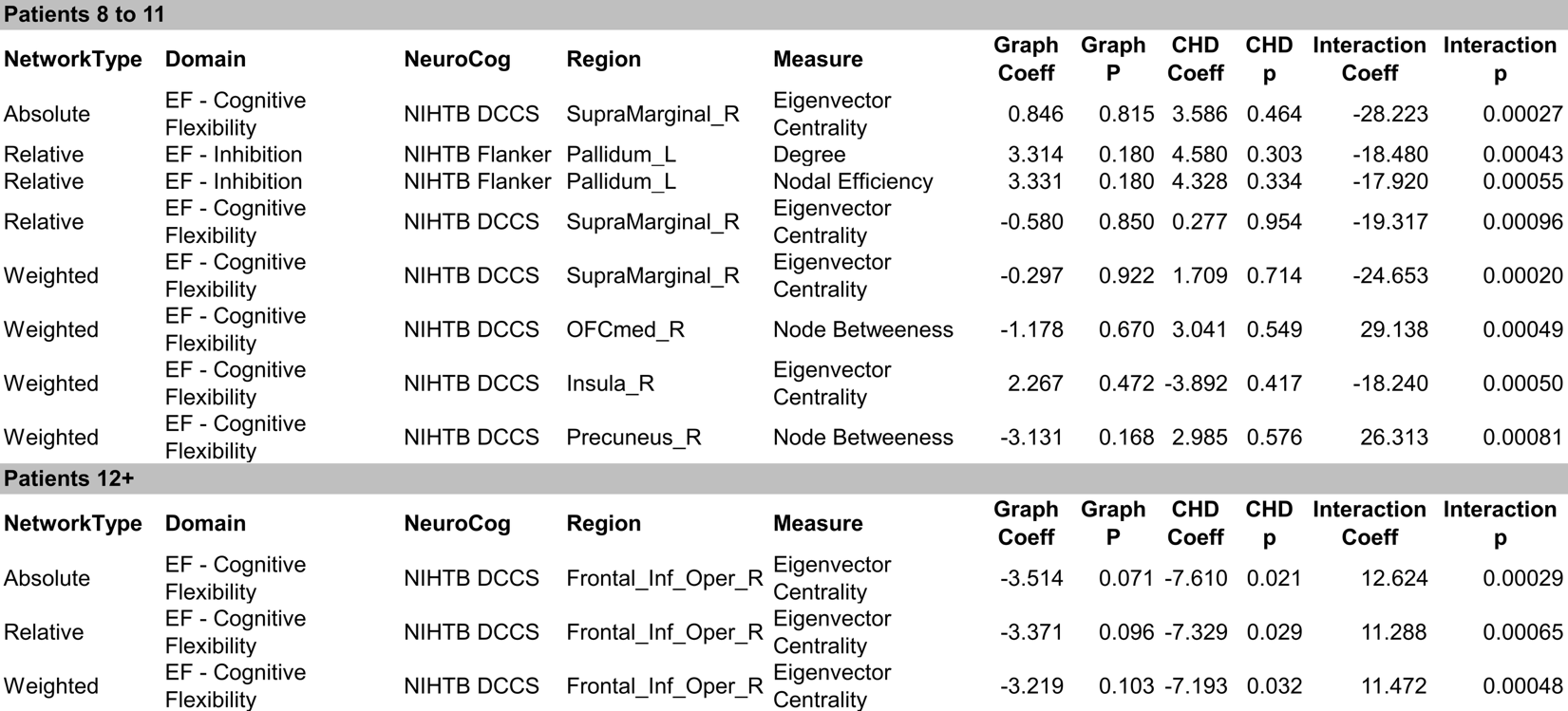


Supplemental Table 4. All results centralized into a single file

Due to the size of the file, it is not included in this document but is uploaded as a separate file: all_results_centralized.csv.
